# Psychiatric disorders and risk of subsequent dementia: Systematic review and meta‐analysis of longitudinal studies

**DOI:** 10.1002/gps.5711

**Published:** 2022-04-23

**Authors:** Jean Stafford, Wing Tung Chung, Andrew Sommerlad, James B. Kirkbride, Robert Howard

**Affiliations:** ^1^ MRC Unit for Lifelong Health and Ageing University College London (UCL) London UK; ^2^ Division of Psychiatry University College London (UCL) London UK; ^3^ Camden and Islington NHS Foundation Trust London UK

**Keywords:** Alzheimer's disease, anxiety, bipolar disorder, dementia, depression, geriatric and old age psychiatry, post‐traumatic stress disorder, psychotic disorders, schizophrenia

## Abstract

**Objectives:**

Although psychiatric disorders have been found to be associated with increased risk of dementia, previous findings are mixed, and the nature of these relationships remains poorly understood. We examined longitudinal associations between depression, anxiety, post‐traumatic stress disorders (PTSD), bipolar disorder (BPD), psychotic disorders and subsequent dementia.

**Methods:**

We searched three databases for longitudinal, population‐based studies investigating associations between psychiatric disorders and dementia (PROSPERO registration: CRD42020209638). We conducted narrative synthesis, and random‐effects meta‐analyses to obtain pooled estimates. We used meta‐regression and stratified analyses to examine variation by sex, age‐at‐onset and follow‐up time.

**Results:**

Fifty‐seven citations met eligibility criteria. Most studies focussed on depression (*n* = 33), which was associated with subsequent all‐cause dementia (pooled relative risk [RR]: 1.96, 95% confidence interval [CI]: 1.59–2.43; *I* ^2^ = 96.5%), Alzheimer's Disease (pooled RR: 1.9, 95% CI: 1.52–2.38; *I* ^2^ = 85.5%), and Vascular Dementia (pooled RR: 2.71, 95% CI: 2.48–2.97; *I* ^2^ = 0). Associations were stronger in studies with shorter follow‐up periods and for severe and late‐onset depression. Findings regarding anxiety were mixed, and we did not find evidence of an overall association (pooled RR: 1.18, 95% CI: 0.96–1.45; *I* ^2^ = 52.2%, *n* = 5). Despite sparse evidence, psychotic disorders (pooled RR: 2.19, 95% CI: 1.44–3.31; *I* ^2^ = 99%), PTSD and BPD were associated with subsequent dementia.

**Conclusions:**

People with psychiatric disorders represent high‐risk groups for dementia, highlighting the importance of ongoing symptom monitoring in these groups. Findings regarding temporality and age‐at‐onset indicate that depression symptoms could reflect prodromal dementia for some individuals. Further longitudinal research is required to determine whether psychiatric disorders represent causal risk factors or early markers of dementia neuropathology.

## Key points

- Psychiatric disorders are associated with increased risk of subsequent dementia.
- Most studies focused on depression, while there was limited longitudinal evidence regarding anxiety, post‐traumatic stress disorder, bipolar disorder or psychotic disorders.
- Severe and late‐onset depression showed stronger associations with dementia than earlier‐onset and mild‐to‐moderate depression.
- Associations between depression and dementia were stronger in studies with shorter follow‐up periods, indicating that depression symptoms could be markers of prodromal dementia for some individuals.

## 1 INTRODUCTION

Psychiatric disorders, including depression, anxiety, post‐traumatic stress disorder (PTSD), and psychotic disorders, have been associated with increased dementia risk.^1^, ^2^, ^3^, ^4^ However, the nature of these associations remains under‐examined and unclear. Improved understanding of relationships between psychiatric disorders and dementia could have important implications for dementia prevention, which is a major priority for public health.

Although the Lancet Dementia Commission identified late‐life depression as one of 12 modifiable risk factors which could be targeted to prevent dementia,^5^ other psychiatric disorders were not identified. This may reflect a scarcity of high‐quality longitudinal evidence, with most previous studies limited by small and unrepresentative samples, and cross‐sectional designs. In addition, previous findings have been mixed, particularly regarding anxiety^6^, ^7^ and PTSD,^8^, ^9^ which may reflect heterogeneity in study characteristics including sample, study design, follow‐up time, and exposure and outcome definition.

The causal direction of the association between psychiatric disorders and dementia remains unclear. Psychiatric symptoms may be risk factors for future dementia or alternatively represent early markers of dementia neuropathology. Most previous studies investigating associations between psychiatric disorders and dementia have only included relatively short follow‐up periods (i.e., <10 years). Dementia's long preclinical period of accumulating neuropathological damage over several decades before cognitive and functional decline are apparent,^10^, ^11^ means that symptoms detected near to dementia diagnosis may represent early dementia symptoms. Therefore, studies with longer follow‐up periods—ideally spanning several decades—are required to determine whether psychiatric disorders represent causal risk factors, or preclinical changes in dementia.

To clarify the nature of these relationships, we synthesised longitudinal evidence on associations between depression, anxiety, PTSD, psychotic disorders, bipolar disorder, and subsequent dementia. We examined whether associations varied by psychiatric disorder and dementia subtype, sex, age‐at‐onset, psychiatric disorder severity, and follow‐up time. We estimated population attributable fractions (PAFs) to indicate the proportion of new dementia cases that would theoretically be preventable through preventing psychiatric disorders in the population.

## 2 METHOD

We followed PRISMA guidelines (Table S1),^12^ including pre‐registering our protocol (http://www.crd.york.ac.uk/PROSPERO, registration number: CRD42020209638).

### 2.1 Search strategy

We searched PubMed, PsycINFO and Web of Science for terms (listed in Table S2) relating to:

- Psychiatric disorders: depression, anxiety, psychotic disorders, bipolar disorder or PTSD
- Dementia: all‐cause dementia, Alzheimer's disease (AD) or vascular dementia (VaD)
- Study design: cohort, case‐cohort or nested case‐control studies

We restricted our search to peer‐reviewed English language papers published in academic journals, with no restrictions on publication dates. We hand‐searched preprint servers (bioRxiv, medRiv and PsyArXiv) and bibliographies of included papers.

Our inclusion criteria were as follows:

- Longitudinal population‐based cohort studies (including case‐cohort or nested case‐control studies). We excluded studies of clinical samples or high‐risk populations, as we judged these to be atypical, with controls unlikely to be representative of the general population.
- Participants aged 18 years or older without dementia or mild cognitive impairment (MCI) at baseline.
- Dementia diagnosed using validated diagnostic criteria.
- Psychiatric disorders meeting diagnostic criteria assessed clinically or via validated screening tool, or attainment of a clinically significant threshold on a validated measure.

### 2.2 Screening and data extraction

Two researchers independently screened 50% of titles and abstracts, and an overlapping randomly selected 10% (inter‐rater agreement: 99.9%, Cohen's *k* = 0.96, *p* = <0.001). Both researchers independently screened all full texts. Discrepancies were resolved by consensus agreement. We extracted data on study characteristics, exposures, outcomes, covariate information, and measures of effect (hazard ratio [HR], incidence rate ratio (IRR), or odds ratio (OR) with 95% CI).

### 2.3 Study quality

One reviewer assessed risk of bias using the Newcastle‐Ottawa Scale.^13^ A second reviewer assessed a randomly selected 10% of citations (inter‐rater agreement: 92.2%, Cohen's *k* = 0.65, *p* = 0.01) and we discussed and resolved discrepancies. We examined small study effects using funnel plots and Egger's test of bias.^14^

### 2.4 Data analysis

We conducted narrative synthesis and meta‐analysis, where five or more comparable citations were available, to examine associations between psychiatric disorders and dementia. Where multiple studies reported analyses of the same cohort, we only included the analysis with a longer follow‐up duration in the meta‐analysis, to avoid duplication and reduce risk of reverse causation bias. We prioritised adjusted effect estimates where possible. We estimated pooled relative risks (RR) using random‐effects meta‐analysis in Stata version 16, which accounts for heterogeneity between studies and allows HRs and ORs to be incorporated into the same analysis.^15^, ^16^ Heterogeneity was assessed using the Q‐test (quantified using I‐squared). Where possible, we conducted a priori subgroup analyses using meta‐regression and stratified analyses to examine variation in associations by sex, follow‐up period, age‐at‐onset, psychiatric disorder severity, and study quality. We estimated PAFs for dementia in relation to each psychiatric disorder using the Levin formula^17^ based on pooled RR estimates and prevalence estimates from the Adult Psychiatric Morbidity Survey (APMS).^18^

## 3 RESULTS

### 3.1 Study characteristics

**FIGURE 1:**
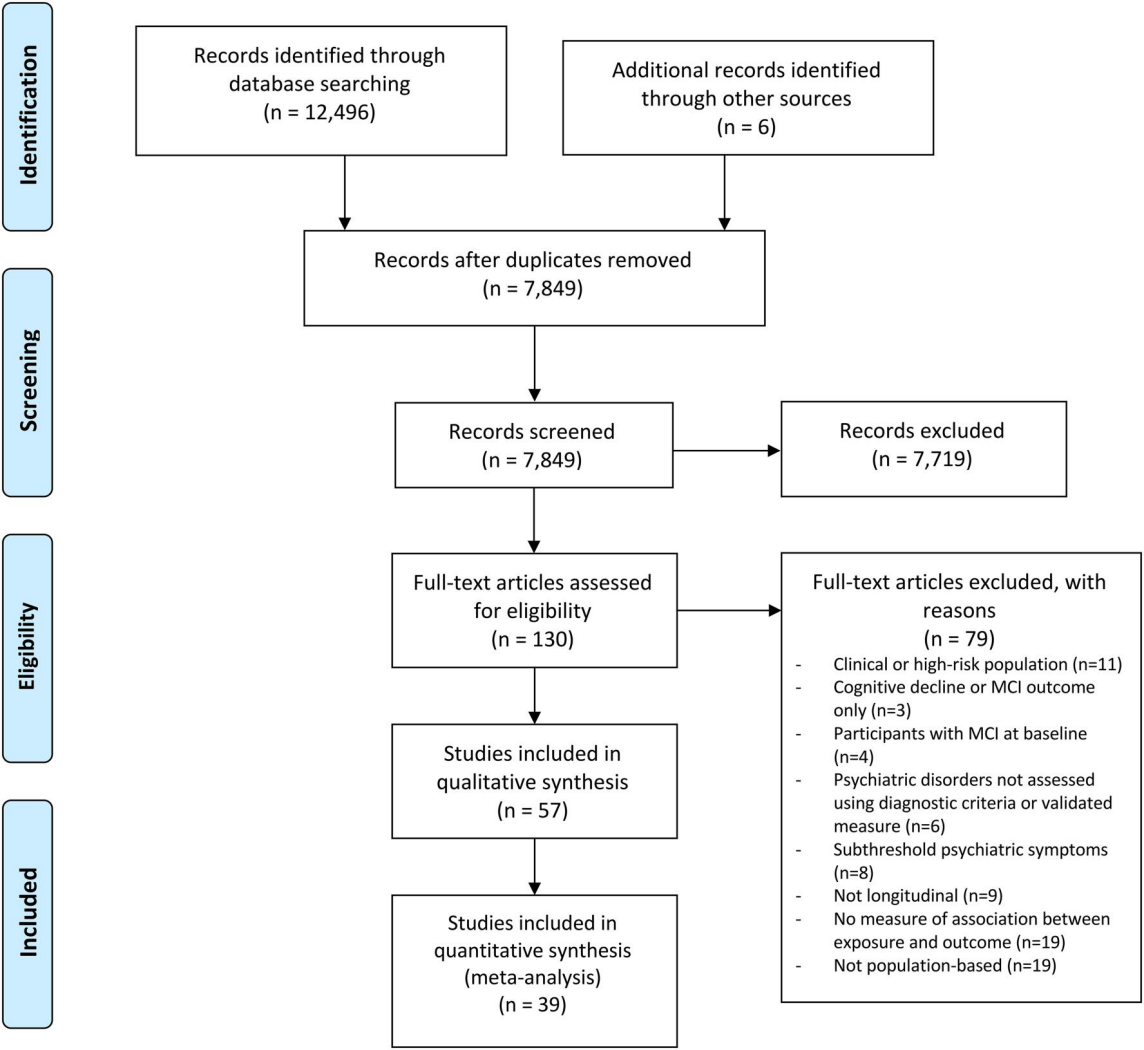
PRISMA flow diagram

We identified 7849 citations, of which 57 met eligibility criteria (Figure 1). Studies were published between 1996^19^ and 2021^20^ (Table 1). Most citations focused on depression (*n* = 33), others involved anxiety (*n* = 6), psychotic disorders (*n* = 7), bipolar disorder (*n* = 4), PTSD (*n* = 3) or mixed conditions (*n* = 4).

**TABLE 1:** Citation characteristics

First author, year (*n*)	Sample, setting and study type	Baseline age (years [SD])^a^	Total follow‐up (years)	Mean (SD) or median (IQR) follow‐up in years^a^	Psychiatric disorder or clinically significant symptoms^b^ ^,^ ^c^	Dementia diagnosis (*N* cases)^c^ ^,^ ^d^	Quality^e^	Summary of findings on association between psychiatric disorder and dementia^f^
Anxiety (*n* = 6)								
de Bruijn 2014	Rotterdam Study, The Netherlands, prospective	Sample 1: 68.6 (8.5), sample 2: 75.5 (6.2)	18	Sample 1: 11.8 (5)Sample 2: 5.8 (1.9)	Sample 1: HADS ≥8	Dementia (DSM‐III‐R, *n* = 358); AD (NINCDS‐ADRDA, *n* = 291)	Good	No evidence of association between anxiety disorder and dementia (HR: 0.81, 95% CI: 0.50–1.30)
Sample 1 *n* = 2708					Sample 2: DSM‐IV			
Sample 2 *n* = 3069								
Gallacher 2009 *n* = 1160	Caerphilly Prospective Study, Wales, prospective	56.1 (4.4)	20	17.3 (1.3)	STAI≥31	Dementia (DSM‐IV, *n* = 69)	Good	Anxiety associated with increased odds of dementia (OR: 2.89, 95% CI: 1.27–6.54)
Mortamais 2018 *n* = 5234	Three‐City (3C) Study, France, prospective	73.4 (5.2)	10	/	STAI≥44	Dementia (DSM‐IV, *n* = 378)AD (NINCDS‐ADRDA, *n* = 259), VaD (NINDS‐AIREN, *n* = 23)	Good	High trait anxiety associated with a higher rate of dementia (HR: 1.26, 95% CI: 1.01–1.57)
Petkus 2016 *n* = 1082	Swedish adoption Twin Study of Ageing, prospective	60.86 (11.15)	28	Years to dementia: 14.65 (6.7)	1 + standard deviation above mean STPI	Dementia (DSM‐III‐R/IV, *n* = 172)	Good	Anxiety associated with increased risk of dementia after adjustment for confounders, including depression (HR: 1.48; 95% CI: 1.01–2.18)
Santabárbara 2019 *n* = 4057	Zaragoza Dementia and Depression study (ZARADEMP), Spain, prospective	No AD: 72.83 (9.03)	4.5	Median: 4.4 (IQR: 3.0–4.9)	GMS AGECAT ≥3	AD (DSM‐IV, *n* = 87)	Poor	AD associated with previous anxiety (SHR: 3.9, 95% CI: 1.59–9.6), but not anxiety ‘subcases’ (SHR: 1.19, 95% CI: 0.75–1.88).
		AD: 83.72 (7.13)						
Santabárbara 2020 *n* = 4057	ZARADEMP, Spain, prospective	VaD (men: 80.3 (8.2), women: 79.8 (7.9)); No VaD (men: 71.7 (9.1), women: 72.3 (9.1))	4.5	Median: 4.4 (IQR: 3.0–4.9)	GMS AGECAT ≥3	VaD (DSM‐IV, *n* = 14)	Poor	Anxiety associated with VaD in men (IRR: 3.24, 95% CI: 1.13–9.35), but not women (IRR: 0.68, 95% CI: 0.19–2.23)
Post‐traumatic stress disorder (*n* = 3)								
Flatt 2018 *n* = 499,844	Kaiser permanente Northern California health system, United States, register‐based	71.1 (7.9)	13	8 (4.6)	ICD‐9: 309.81	Dementia (ICD‐9, *n* = 59,127)	Good	PTSD associated with higher rate of dementia (female HR: 1.59, 95% CI: 1.3–1.95; male HR: 1.96, 95% CI: 1.51–2.55)
Gradus 2019 *n* = 279,188	Danish population register	51	17	Stress cohort median: 6.1; Comparison: 6.8	Stress disorders: ICD‐10: F43.x	Dementia (ICD‐10, stress cohort *n* = 1364)	Good	Those with PTSD had 2 times the rate of dementia compared to those without PTSD (95% CI: 1.3–3.2)
Wang 2016 *n* = 8750	Nationwide Health Insurance Research Database (NHIRD), Taiwan, register‐based	PTSD: 55.44 (9.20)No PTSD: 55.42 (9.22)	11	Years to dementia: PTSD: 4.68 (2.31); No PTSD: 6.96 (2.66)	ICD‐9‐CM: 309.81	Dementia (ICD‐9‐CM, *n* = 135)	Good	PTSD (HR: 4.37, 95% CI: 2.53–7.55) and depression (HR: 2.16, 95% CI: 1.28–3.66) associated with subsequent dementia, with a dose‐response relationship by PTSD severity
Non‐affective psychotic disorder (*n* = 7)								
Almeida 2018 *n* = 38,173	Health in Men Study (HIMS), Western Australia, prospective	72.5 (4.6)	17.7	/	Non‐affective psychotic disorders: ICD‐8/9: 295, 297; ICD‐10: F20, F22, F23, F25, F28, F29	Dementia (ICD‐8/9/10, *n* = 8068)	Good	Psychotic disorders associated with subsequent dementia (SHR: 2.67, 95% CI: 2.3–3.09). Stronger association for shorter duration of psychosis. No variation by age‐at‐onset
Kodesh 2020 *n* = 94,120	Health maintenance organisation data, Israel, register‐based	68.9 (7.1)	4.83	/	VLOSLP: ICD‐9: 295–299; ICD‐10: F20–F29	Dementia (ICD‐9/10, *n* = 6026)	Good	Very late‐onset schizophrenia associated with subsequent dementia (HR: 2.67, 95% CI: 1.82–3.91).
Kørner 2009 *n* = 12,616	Danish population register	Median LOS: 53.56, comparison: 65.13, VLOSLP and comparison: 71.19	4.58	Median (IQR) LOS: 3.15 (1.56–3.50)VLOSLP: 3 (1.25–4.78)	LOS and VLOSLP: ICD‐10: F20‐F20.9	Dementia (ICD‐10, LOS *n* = 20, comparison *n* = 160; VLOSLP *n* = 18, comparison *n* = 157).	Good	LOS and VLOSLP had higher dementia rates than osteoarthritis patients (LOS rate ratio (RR): 3.47, 95% CI: 2.19–5.5; VLOSLP RR: 3.15, 95% CI: 1.93–5.14) and general population (LOS RR: 2.36, 95% CI: 1.54–3.62; VLOSLP RR: 2.21, 95% CI: 1.39–3.5).
Lin 2018 *n* = 30,200	NHIRD, Taiwan, register‐based	/	14	Schizophrenia: 9.4 (11.8), control: 9.5 (10.1)	Schizophrenia: ICD‐9‐CM: 295	Dementia (ICD‐9‐CM, *n* = 1237), AD (ICD‐9‐CM, *n* = 147), VaD (ICD‐9‐CM, *n* = 162)	Good	Patients with schizophrenia had higher rates of dementia (HR: 2.01, 95% CI: 1.42–2.59), AD (HR: 2.27, 95% CI: 1.99–3.51), and VaD (HR: 2.01, 95% CI: 1.36–2.22) than controls
Ribe 2015 *n* = 2,845,440	Danish population register	58.7 (11.2)	18	11 (6)	Schizophrenia: ICD‐8/9: 295 (except 295.79); ICD‐10: F20; Schizoaffective disorder: ICD‐8/9: 295.79, 296.8; ICD‐10: F25	Dementia (ICD‐8/9/10, *n* = 136,012)	Good	Schizophrenia associated with incident dementia (IRR: 1.71, 95% CI: 1.6–1.82)
Stafford 2021 *n* = 169,499	Swedish population register	70.31 (7.2)	30	8.6 (6.68)	VLOSLP: ICD‐10: F20‐F29 (or ICD‐8/9 equivalent)	Dementia (ICD‐8/9/10, *n* = 13,610)	Good	VLOSLP associated with higher rate of dementia (HR: 4.22, 95% CI: 4.05–4.41). Association attenuated over time but was present for up to 20 years
Stroup 2021 *n* = 8,011,773	Medicare beneficiaries, US, register‐based	74 (8.2)	11	/	Schizophrenia: ICD‐8/9/10: F25	Dementia (ICD‐9/10, *n* = 1,129,646)	Good	Schizophrenia associated with elevated rate of dementia (age 66 dementia rate – schizophrenia: 52.5 (95% CI: 50.1–54.9), controls: 4.5 (95% CI: 4.4–4.6) per 1000 person‐years at‐risk
Bipolar disorder (BPD) (*n* = 4)								
Almeida 2018 *n* = 38,173	Western Australian Data Linkage System, register‐based	72.5 (4.6)	17.6	12.8 (5.3)	ICD‐8: 296.1, 296.3; ICD‐9: 296.0, 296.1, 296.4, 296.5, 296.6, 296.7, 296.80, 296.81; ICD‐10: F30, F31	Dementia (ICD‐8/9/10, *n* = 423)	Good	Late‐onset (≥60 years HR: 2.99, 95% CI: 2.17–4.12) and younger onset BPD associated with dementia (<60 years HR: 2.31, 95% CI: 1.77–3.01)
Almeida 2016 *n* = 37,768	HIMS, Western Australia, prospective	72.5 (4.6)	13	/	ICD‐8: 296.1 and 296.3; ICD‐9: 296.0, 296.1, 296.4, 296.5, 296.6, 296.7, 296.80, 296.81	Dementia (ICD‐8/9, *n* = 4925)	Good	BPD associated with higher dementia rates (HR: 2.3, 95% CI: 1.80–2.94). Stronger association for shorter duration of BPD, or illness onset after 70 years old
Lin 2020 *n* = 102,675	NHIRD, Taiwan, register‐based	BPD: 55.31 (8.48), comparison: 55.25 (8.55)	9	BPD: 6.56 (2.97); comparison: 6.86 (2.85)	ICD‐9‐CM: 296 except 296.2x, 296.3x, 296.9x, and 296.82	Dementia (ICD‐9‐CM, *n* = 2122), AD (ICD‐9‐CM, *n* = 1353), VaD (ICD‐9‐CM, *n* = 447)	Good	BPD associated with incident dementia (HR: 7.52, 95% CI: 6.86–8.25), AD (HR: 13.16, 95% CI: 11.58–14.96) and VaD (HR: 5.5, 95% CI: 4.53–6.69)
Wu 2013 *n* = 64,804	NHIRD, Taiwan, register‐based	74.1 (8.6)	9	/	ICD‐9‐ CM: 296.0x, 296.1x, 296.4x, 296.5x, 296.6x, 296.7x, 296.80, 296.81, 296.89	Dementia (ICD‐9‐CM, *n* = 9304)	Good	BPD associated with increased odds of dementia (OR: 4.32, 95% CI: 3.21–5.82)
Multiple psychiatric diagnoses (*n* = 4)								
Chen 2015 *n* = 4582	NHIRD, Taiwan, register‐based	Control: 65.34 (7.47); major depressive disorder: 65.45 (7.53); BPD: 64.72 (7.08)	13	Years to dementia: controls: 5.62 (3.23), MDD: 4.01 (3.02); BPD: 4.38 (3.60)	MDD: ICD‐9‐CM: 296.2x, 296.3x; BPD: ICD‐9‐CM: 296.0x, 296.1x, 296.4x, 296.5x, 296.6x, 296.7x, 296.80, 296.81, 296.89	Dementia (ICD‐9‐CM, *n* = 547)	Good	BPD (HR: 5.58, 95% CI: 4.26–7.32) and MDD (HR: 3.02, 95% CI: 2.46–3.7) associated with higher rates of dementia
Kessing 1999 *n* = 13,852	Danish population register	Neurosis: 41.2 (13.8), depression: 51.3 (16.0), schizophrenia: 31.7 (15.4)	24.7	Median: 21.6	Depressive episode: ICD‐8: 296.09, 296.29, Schizophrenia: ICD‐8: 295, Neurosis: ICD‐8: 300	Dementia (ICD‐8/9/10, *n* = 424)	Poor	Rates of dementia were increased 14.7‐fold (95% CI: 9.1–22.4) in those with schizophrenia, 13.7‐fold (95% CI: 12.1–15.4) for affective disorder, and 11.2‐fold (95% CI: 9.6–12.9) for neurosis
Tapiainen 2017 *n* = 55,896	Medication use and Alzheimer's disease study, Finland, register‐based nested case‐control	79.7 (6.8)	33	Time to AD: AD cases: 18.1 years (8.9), controls: 19.4 (9.0)	Psychotic disorders: ICD‐10: F20–F29; mood disorders: ICD‐10: F32–F39; neurotic disorders: ICD‐10: F40–F48	AD (NINCDS‐ADRDA and DSM‐IV, *n* = 27,948)	Good	Depression associated with increased odds of AD with 5‐year (OR: 1.17, 95% CI: 1.05–1.3), but not 10‐year interval between diagnoses (OR: 1.08, 95% CI: 0.96–1.23). Anxiety and psychotic disorders were not associated with AD regardless of interval
Zotcheva 2018 *n* = 28,916	The Nord‐Trøndelag Health Study, Norway, prospective	Midlife moderate‐to‐vigorous physical activity (MVPA): 52.3,No MVPA: 54.6	16.3	15.2	Psychological distress: ADI‐4 ≥88th percentile	Dementia (ICD‐10, *n* = 920)	Good	Psychological distress associated with higher dementia rates (HR: 1.34, 95% CI: 1.03–1.74)
Depression (*n* = 33)								
Almeida 2017 *n* = 4922	HIMS, Western Australia, prospective	77.2 (3.7)	14.3	8.9	Short version GDS‐15 score ≥7	Dementia (ICD‐8/9/10, *n* = 903)	Good	Depression associated with dementia (past depression SHR: 1.3, 95% CI: 1.0–1.6, baseline SHR: 1.5, 95% CI: 1.2–2.0)
Becker 2009 *n* = 288	Cardiovascular Health Study‐Cognition Study, US, prospective	77.52 (3.65)	9	7.1	CES‐D score ≥10	Dementia (DSM‐IV, *n* = 48)	Poor	No evidence of association between persistent depression and dementia (HR: 1.33, 95% CI: 0.49–3.65)
Berger 1999 *n* = 222	The Kungsholmen Project, Sweden, prospective	Incident AD: 85.53 (4.95); no dementia: 83.18 (4.77)	3	3.08 (0.58)	CPRS	AD (DSM‐III‐R, *n* = 34)	Poor	Depression associated with subsequent AD, particularly for motivation‐related symptoms (OR: 1.4, 95% CI: 1.04–1.89)
Buntinx 1996 *n* = 19,103	Family‐practice‐based registration network, The Netherlands, register‐based	50+	10	/	ICPC‐classification: P76	Dementia (ICPC: *n* = 137)	Poor	Depression among older people associated with increased risk of dementia (HR: 2.55, 95% CI: 1.19–5.47)
Chan 2020 *n* = 16,725	NHIRD, Taiwan, register‐based	41.5 (15.9)	12	Time to dementia: control: 10.71 (1.16); MDD: 10.45 (1.86)	ICD‐9‐CM: 296.2x and 296.3x	Dementia (ICD‐9‐CM, *n* = 508), AD (ICD‐9‐CM, *n* = 458)	Good	Patients with MDD had increased risk of dementia and AD. Risk highest among difficult‐to‐treat patients
Chen 2008UK *n* = 3,341,China *n* = 1254	MRC–Ageing in Liverpool Project Health Aspects (MRC‐ALPHA), UK; Hefei cohort, China	65+	MRC‐alpha: 2‐4Hefei‐ 1	/	GMS‐AGECAT score ≥3	Dementia (GMS‐AGECAT: Hefei *n* = 75, MRC‐ALPHA, *n* = 382	Poor	Incident dementia associated with severe, but not milder, depression (Hefei cohort HR: 5.44, 95% CI: 1.67–17.8; MRC‐ALPHA, 4‐year follow‐up HR: 2.62, 95% CI: 1.18–5.8).
Deckers 2018 *n* = 278	The Cambridge City over‐75s cohort (CC75 C), UK, prospective	87.8 (3.1)	18	/	CAMDEX Depressive Symptoms Scale score ≥6	Dementia (CAMDEX, post‐mortem, cause of death, *n* = 76)	Good	Depression not associated with incident dementia (OR: 0.78, 95% CI: 0.34–1.78).
Ezzati 2019 *n* = 1219	The Einstein Ageing Study, US, prospective	78.3 (5.3)	17.2	4.5 (3.5)	GDS score ≥6	Dementia (DSM‐IV, *n* = 132)	Good	Depression associated with dementia in longer‐term (>3 years HR: 1.13, 95% CI: 1.01–1.26), but not short‐term follow‐up (<3 years HR: 1.09, 95% CI: 0.99–1.2).
Gatz 2005 *n* = 766	Manitoba Study of Health and Ageing, Canada, prospective	74.5 (6)	5	/	CES‐D score ≥16	Dementia (DSM‐III‐R, *n* = 56), AD (NINCDS‐ADRDA, *n* = 36)	Poor	Depression associated with dementia (CES‐D ≥16 HR: 2.37, 95% CI: 1.02–5.54) and AD (OR: 2.75, 95% CI: 1.04–7.24).
Geerlings 2000 *n* = 3147	Amsterdam Longitudinal Study of the Elderly, The Netherlands, prospective	Depression: 73.6 (5.7), no depression: 73.7 (5.7)	4	3.2	GMS‐AGECAT score ≥3	AD (DSM‐III‐R, *n* = 53)	Good	Depression associated with increased risk of AD (OR: 1.67, 95% CI: 0.76–3.63).
Gracia‐Garcia 2015 *n* = 3864	ZARADEMP, Spain, prospective	Depression: 73.6 (9.3),No depression: 71.5 (8.9)	4.5	/	GMS‐AGECAT score ≥3	AD (DSM‐IV, *n* = 70)	Poor	Severe depression associated with subsequent AD (SHR: 4.3, 95% CI: 1.39–13.33).
Heser 2013 *n* = 2663	German Study on Ageing, Cognition, and Dementia in Primary Care Patients, prospective	81.2	8	/	CIDI	Dementia (DSM‐IV and ICD‐10, *n* = 308), AD (DSM‐IV, *n* = 152)	Good	Very late‐onset depression and current depressive symptoms both predicted all‐cause dementia
Heser 2020 *n* = 97,110	German health insurance provider, Allgemeine Ortskrankenkasse	74.7 (6.6)	9	5.82	ICD‐10: F32	Dementia (ICD‐10, *n* = 20,779)	Good	Depression associated with dementia (IRR: 1.58, 95% CI: 1.51–1.64). Stronger association found for shortest interval, men, and younger participants
Holmquist 2020 *n* = 3,341,010	Swedish population register	Matched cohort: 63.79 (11.89); Sibling cohort‐ depression: 59.10 (8.85), no depression: 59.97 (8.91)	35	10.41 (6.89)	ICD‐10: F32, F33ICD‐8/9: 311, 296B	Dementia (ICD‐8/9/10, *n* = 9802), AD (ICD‐8/9/10, n = 4201), VaD (ICD‐8/9/10, *n* = 2329)	Good	Depression associated with dementia (OR: 2.47, 95% CI: 2.35–2.58), VaD (OR: 2.68, 95% CI: 2.44–2.95), and AD (OR: 1.79, 95% CI: 1.68–1.92). Association strongest in first 6 months after depression diagnosis but persisted for more than 20 years
Irie 2008 *n* = 1932	Honolulu‐Asia Ageing Study, Japan, prospective	76.3 (3.6)	6	/	CES‐D score ≥9	Dementia (DSM‐III‐R, *n* = 98)	Good	Depression associated with higher rate of dementia (HR: 2.2, 95% CI: 1.3–3.7) and AD (HR: 2.9, 95% CI: 1.4–5.9), but not VaD (HR: 1.3, 95% CI: 0.3–5.8)
Karlsson 2015 *n* = 2404	The Swedish Twin Registry	Dementia: 80.1 (6.6), no dementia: 78.9 (6.6)	>10 years	/	ICD‐7: 314.99; ICD‐8: 296.00, 298.00, 300.40–41, 790.20; ICD‐9: 296 C/D/W, 298A, 300E, 309 A/B, 311X; ICD‐10: F32, F33, F34.1, F41.2; Self‐report or anti‐depressant use; CES‐D score ≥20	Dementia (DSM‐III‐R/IV, *n* = 804), AD (NINCDS‐ADRDA, *n* = 469)	Good	Depression associated with increased risk of dementia (HR: 3.41, 95% CI: 2.72–4.27). Strongest association in the 10 years before dementia diagnosis and for late‐onset depression
Köhler 2015 *n* = 35,791	The Dutch Registration of Family Practices, Limburg, The Netherlands, register‐based	65+	13	/	ICPC code: P76	Dementia (ICPC, *n* = 1680)	Good	Depression associated with increased risk of dementia (HR: 2.03, 95% CI: 1.56–2.64)
Köhler 2011 *n* = 771	The Maastricht Ageing Study, The Netherlands, prospective	67.1 (7.3)	9	/	Scores in upper quartile of SCL‐90 (revised version).	Dementia (DSM‐III‐R/IV, *n* = 37), AD (NINCDS‐ADRDA, *n* = 26), VaD (NINDS‐AIREN, *n* = 11)	Fair	Depression associated with all‐cause dementia (OR: 2.06, 95% CI: 1.01–4.22), but not AD (OR: 1.81, 95% CI: 0.78–4.23) or VaD (OR: 3.03, 95% CI: 0.86–10.64)
Kontari 2019 *n* = 4589	English Longitudinal Study of Ageing, prospective	50+	10	/	CES‐D score ≥4	Dementia (participant or informant reported physician diagnosed dementia, and/or IQCODE score ≥3.5, *n* = 216)	Poor	Depression associated with higher rate of dementia during follow‐up (HR: 1.82, 95% CI: 1.13–2.95), with attenuation after adjusting for baseline cognitive function (HR: 1.28, 95% CI: 0.78–2.08)
Lenoir 2011 *n* = 7989	The 3C Study, France, prospective	74 (5.4)	4	/	Self‐reported lifetime treated depression. MDD (DSM‐IV). CES‐D baseline score ≥22 (women), ≥16 (men)	Dementia (DSM‐IV, *n* = 276), AD (NINCDS‐ADRDA, *n* = 180), VaD (NINDS‐AIREN, *n* = 24)	Poor	Dementia associated with baseline depressive symptoms (HR: 1.5, 95% CI: 1.2–2.2), but not self‐reported treated depression or diagnosed MDD
Li 2011 *n* = 3410	Adult Changes in Thought Study, US, prospective	Significant depressive symptoms: 75.8 (6.2),No depression: 74.8 (6.2)	15	7.1	CES‐D score ≥16; self‐reported history of depression	Dementia (DSM‐IV, *n* = 658)	Good	Baseline depression associated with dementia (HR: 1.71, 95% CI: 1.37–2.13), for late‐life (age‐at‐onset ≥50 years) (HR: 1.46, 95% CI: 1.16–1.84), but not early‐life depression (age‐at‐onset <50 years) HR: 1.1, 95% CI: 0.83–1.47)
Lin 2017 *n* = 49,955	NHIRD, Taiwan, register‐based	Median: 39, IQR: 29–51	10	Depression median: 7.19 (IQR: 5.95–8.48), control: 7.22 (IQR: 6.01–8.51)	ICD‐9‐CM: 296.2x–296.3x, 300.4, 311.x	VaD (ICD‐9‐CM, *n* = 117)	Good	Depression associated with higher rate of VaD (HR: 3.1, 95% CI: 2.13–4.52)
Luppa 2013 *n* = 1265	Leipzig Longitudinal Study of the Aged, Germany, prospective	81.5	8	4.3 (2.4)	CES‐D score ≥23; MDD diagnosis in DSM‐III‐R	Dementia (DSM‐III‐R/‐IV and ICD‐10, *n* = 183)	Good	Major depression diagnosis associated with higher rate of dementia (HR: 2.75, 95% CI: 1.01–7.5)
Mirza 2014 *n* = 4393	Rotterdam Study, The Netherlands, prospective	72.7 (7.3)	13.7	8.7 (3.5)	CES‐D score ≥16	Dementia (DSM‐III‐R, *n* = 582), AD (NINCDS‐ADRDA, *n* = 489)	Good	Depression associated with dementia in short (HR: 1.08, 95%CI: 1.00–1.17) (0–5 years HR: 1.13, 95%CI: 1.01–1.27) and intermediate (5–10 years HR: 1.14, 95%CI: 1.01–1.29), but not long‐term follow‐up (>10 years HR: 0.83, 95%CI: 0.66–1.04)
Richard 2013 *n* = 1483	Washington Heights–Inwood Columbia Ageing Project, US, prospective	Depression: 77.7, (7.2), No depression: 76.7 (7.0)	10.1	5.4	CES‐D score ≥4	Dementia (DSM‐III‐R, *n* = 207), AD (NINCDS‐ADRDA, *n* = 164), VaD (NINDS‐AIREN, *n* = 33)	Good	Baseline depression associated with higher rate of dementia (HR: 1.8, 95%CI: 1.2–2.7), and AD (HR: 1.9, 95%CI: 1.2–2.9), but not VaD (HR: 1.7, 95% CI: 0.5–5.6)
Rolandi 2020 *n* = 1100	Brain ageing in Abbiategrasso (InveCe.Ab), Italy, prospective	70–74	8	7	DSM‐IV‐TR MDD or dysthymiaOR 3+: (i) depression history, (ii) depression treatment, (iii) GDS score ≥8, (iv) depressed mood in last week.	Dementia (DSM‐IV‐TR, *n* = 111)	Poor	Depression not associated with subsequent dementia (SHR: 0.99, 95% CI: 0.4–2.45)
Saczynski 2010 *n* = 949	The Framingham Heart Study, US prospective	79 (5)	17	8	CES‐D score ≥16	Dementia (DSM‐IV, *n* = 164)AD (DSM‐IV, *n* = 136)	Good	Depression associated with increased risk of dementia (HR: 1.72, 95% CI: 1.04–2.84) and AD (HR: 1.76, 95% CI: 1.03–3.01)
Singh‐Manoux 2017 *n* = 10,308	Whitehall II, UK, prospective	1985‐ Depression: 44.5 (6), no depression: 45.1 (6); 2003‐ Depression: 60 (6), no depression: 61.5 (6)	28	1985: 26.6 (4.5)1991: 21.7 (3.6)1997: 16.3 (2.7),2003: 11.1 (1.8)	General Health Questionnaire (GHQ‐30) ≥5CES‐D score ≥16	Dementia (ICD‐10, *n* = 322)	Good	Dementia associated with depression in late (mean follow‐up 11 years) (HR: 1.67, 95% CI: 1.11–2.49, but not early study phase (mean follow‐up 22 years) (HR: 1.02, 95% CI: 0.72–1.44)
Spira 2012 *n* = 302	Study of Osteoporotic Fractures, US, prospective	86.9 (2.1)	5	/	GDS score ≥6	Dementia (DSM‐IV‐R, *n* = 84)	Poor	Depression associated with increased risk of dementia (OR: 3.15, 95% CI: 1.03–9.65)
Tao 2019 *n* = 8880	NHIRD, Taiwan, register‐based	71.55 (5.47)	7	6.94 (0.5)	ICD‐9‐CM: 296.2x at least 2x in 6 months and prescribed antidepressant medication for 90 days and 6 months + after initial diagnosis.	AD (ICD‐9‐CM, *n* = 155)	Good	Depression associated with increased risk of AD (HR: 2.21, 95% CI: 1.57–3.31)
Vilalta 2013 *n* = 451	Estudio de Verona, Spain, prospective	76.9 (5.5)	5	/	DSM‐IV diagnosis of major depression	Dementia (DSM‐IV, *n* = 52), AD (DSM‐IV, *n* = 30)	Poor	Late‐onset depression associated with increased risk of dementia (HR: 2.64, 95% CI: 1.15–6.0)
Wallin 2013 *n* = 212	The Umeå 85+/GERDA (GErontologisk Regional DAtabas), Sweden, prospective	Dementia: 88.54 (3.7), no dementia 88.92 (4.35)	7	Dementia: 3.82 (1.22) no dementia: 3.24 (1.71)	DSM‐IV diagnosis of depression	Dementia (DSM‐IV, *n* = 71)	Good	Baseline (OR: 2.91, 95% CI: 1.37–6.16) and follow‐up depression (OR: 1.61, 95% CI: 1.26–2.05) associated with dementia
Wu 2020 *n* = 16,210	Survey of Health, Ageing and Retirement in Europe (SHARE), 14 countries in Europe	70.13	10	7.98 (2.61)	Europe‐depression (EURO‐D) scale score ≥4	Dementia (participant‐or proxy‐reported physician diagnosis, *n* = 1030)	Good	Late‐life depression associated with dementia (SHR: 1.52, 95% CI: 1.32–1.75), although only in those below age 80 years. Dose‐response relationship found between depression severity and dementia risk (*p* for trend <0.001)

^a^ Standard deviation (SD), interquartile range (IQR).

^b^ Anxiety and Depression Index (ADI‐4), Center for Epidemiologic Studies‐Depression (CES‐D), Composite International Diagnostic Interview (CIDI), Comprehensive Psychopathological Rating Scale (CPRS), Geriatric Depression Scale (GDS‐15), Hospital Anxiety and Depression Scale (HADS), Spielberger State Trait Anxiety Inventory (STAI), State‐Trait Personality Inventory (STPI), Symptom Checklist‐90‐Revised (SCL‐90).

^c^ International Classification of Health Problems in Primary Care (ICPC), The Geriatric Mental State ‐ Automated Geriatric Examination for Computer Assisted Taxonomy (GMS‐AGECAT), Cambridge Diagnostic Examination for the Elderly (CAMDEX).

^d^ Alzheimer's disease (AD), Vascular dementia (VaD).

^e^ Newcastle‐Ottawa Scale for cohort studies.

^f^ Hazard ratio (HR), incidence rate ratio (IRR), odds ratio (OR), subdistribution hazard ratio (SHR).

Sample sizes ranged from 212^21^ to 8,011,773^22^ (median: 5,234, interquartile range [IQR]: 1483–37,768). 21 studies were register‐based, while 36 involved prospective cohorts. Study quality was assessed as good (*n* = 42, 74%), fair (*n* = 1, 1%), or poor (*n* = 14, 25%). Follow‐up periods ranged from 1^23^ to 35 years^24^ (median: 12 years, IQR: 8–17.7), and 29 of 57 studies (50.9%) had maximum follow‐up periods longer than 10 years.

### 3.2 Depression

Thirty‐nine citations reported data on associations between depression and subsequent dementia (Table 1), with studies conducted in Australia, Asia, Europe, and North America. Follow‐up periods ranged from 1^23^ to 35 years,^24^ and sample sizes were between 212^21^ and 3,341,010 individuals.^24^ Quality was assessed as good (*n* = 27), fair (*n* = 1) or poor (*n* = 11). Depression was indexed via ICD or DSM criteria, International Classification of Health Problems in Primary Care, GMS‐AGECAT, validated scale or screening tool, or a combined approach (Table 1).

**FIGURE 2:**
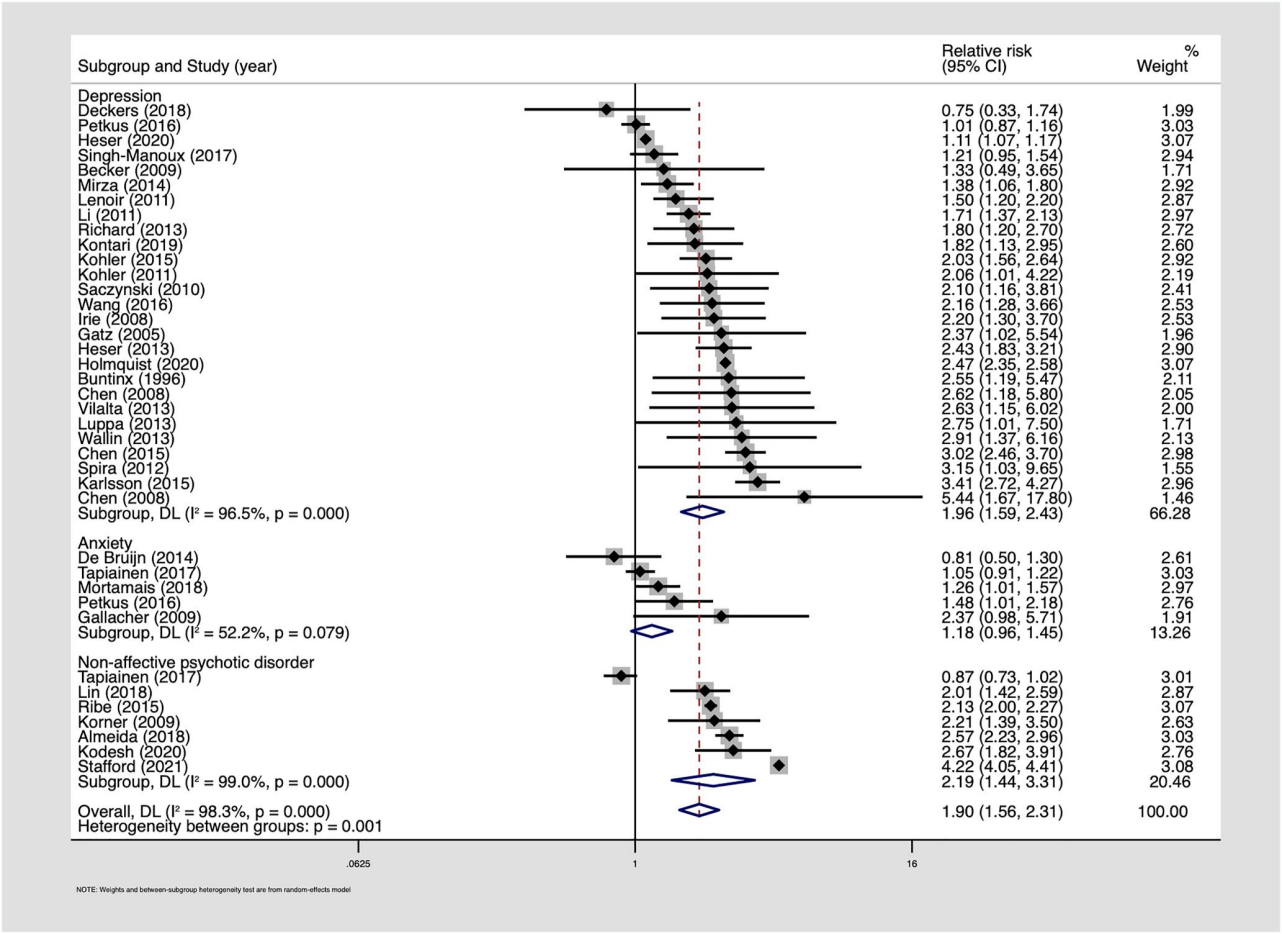
Forest plot—longitudinal associations between depression, anxiety, non‐affective psychotic disorders and subsequent dementia

Depression was associated with increased risk of all‐cause dementia in 30 of 33 studies examining this relationship, while only 3 prospective cohort studies conducted in the United Kingdom, Italy and the United States, found no association.^25^, ^26^, ^27^ Quantitative synthesis of 27 comparable estimates from 26 citations demonstrated an association between depression and incident all‐cause dementia (pooled RR: 1.96, 95% CI: 1.59–2.43; *I* ^2^ = 96.5%, Figure 2). We did not find evidence of small study effects from visual inspection of a funnel plot (Figure S1) or Egger's test of bias (*p* = 0.45).

Similarly, 15 of 17 studies examining depression and subsequent AD reported associations with only two prospective cohort studies finding no association.^28^, ^29^ Based on 13 comparable estimates, we found evidence of a longitudinal association between depression and AD (pooled RR: 1.90, 95% CI: 1.52–2.38; *I* ^2^ = 85.5%, Figure S2). Findings regarding depression and VaD were mixed, with register‐based studies in Taiwan^30^ and Sweden,^24^ and a prospective cohort study in France^29^ finding associations, whereas prospective cohort studies in the United States,^31^ The Netherlands,^28^ and Japan^32^ did not observe associations. In a meta‐analysis of six estimates, we found an overall association between depression and VaD (pooled RR: 2.71, 95% CI: 2.48–2.97; *I* ^2^ = 0, Figure S3). As above, we did not find evidence of small study effects (Egger's test: AD *p* = 0.6, VaD *p* = 0.5).

### 3.3 Subgroup analyses

Using random‐effects meta‐regressions, we found no evidence that associations between depression and all‐cause dementia varied by sex, study quality, study type or age‐at‐onset (Table 2, Figures S4–S6), although in stratified analyses we found an association between dementia and late‐onset depression (>60/65 years; pooled RR: 1.92, 95% CI: 1.13–3.24; *I* ^2^ = 87.8%; five estimates), but not early or mid‐life depression (<60/65 years pooled RR: 1.17, 95% CI: 0.8–1.73; *I* ^2^ = 54.6%; six estimates, Table 2, Figure S7). We found a weaker association between depression and dementia in studies with longer (≥15 years: pooled RR: 1.46, 95% CI: 0.94–2.28; *I* ^2^ = 97.1%; meta‐regression *p* = 0.04) compared with studies with shorter follow‐up periods (≤8 years pooled RR: 2.15, 95% CI: 1.81–2.55; *I* ^2^ = 7.3%, Table 2, Figure S8). We observed no differences between studies with medium (9–14 years pooled RR: 1.98, 95% CI: 1.36–2.89; *I* ^2^ = 95.5%; meta‐regression *p* = 0.40) and shorter follow‐up periods.

**TABLE 2:** Association between depression and all‐cause dementia: stratified analyses and meta‐regression

Analysis	N estimates	Pooled RR (95% CI)	Heterogeneity (I2)	Meta‐regression RR (95% CI)
All‐cause dementia	27	1.96 (1.59–2.43)	96.5%	‐
Dementia subtype				
AD	13	1.9 (1.52–2.38)	85.5%	‐
VaD	6	2.71 (2.48–2.97)	0%	‐
Sex				0.88 (0.57–1.36)
Male	5	2.15 (1.7–2.71)	86.8%	
Female	5	1.92 (1.34–2.75)	97.7%	
Age‐at‐onset				1.65 (0.81–3.37)
Late‐onset	5	1.92 (1.13–3.24)	87.8%	
Early or mid‐life onset	6	1.17 (0.8–1.73)	54.6%	
Follow‐up				
1–8 years (reference)	11	2.15 (1.81–2.55)	7.3%	
9–14 years	10	1.98 (1.36–2.89)	95.5%	0.85 (0.57–1.26)
>15 years	6	1.46 (0.94–2.28)	97.1%	0.64 (0.42–0.99)
Quality				0.85 (0.57–1.28)
Fair or good	18	1.87 (1.45–2.41)	97.7%	
Poor	9	1.95 (1.56–2.45)	8.4%	
Study type				1.10 (0.76–1.61)
Register‐based	6	2.10 (1.32–3.33)	99.2%	
Prospective cohort	21	1.90 (1.53–2.36)	83%	

Abbreviations: CI, confidence intervals. RR, relative risk

We also found narrative evidence for stronger associations when intervals between depression and dementia were shorter. In Swedish register data, the association with dementia was strongest in the first 6 months after depression diagnosis (OR: 20.85, 95% CI: 9.63–45.12), then decreased rapidly but persisted for over 20 years (OR: 2.33, 95% CI: 1.32–4.11).^24^ In Finnish register data, depression and AD were associated during a 5‐year (OR: 1.17, 95% CI: 1.05–1.3), but not 10‐year interval between diagnoses (OR: 1.08, 95% CI: 0.96–1.23).^33^ In Swedish register data, the association was strongest in the 10 years before dementia diagnosis (HR: 4.46, 95% CI: 3.44–5.76), compared with >10 years prior (HR: 1.58, 95% CI: 1.07–2.34).^34^ Prospective cohort studies in the Netherlands and Australia also found associations in short‐term (<5 years), but not longer‐term follow‐up (>10 years).^35^ In Whitehall II data with 28 years of follow‐up, differences in depressive symptoms only emerged around 11 years before dementia diagnosis, and were not observed in longer‐term follow‐up.^36^

We found narrative evidence of stronger associations between dementia and severe, relative to mild or moderate, depression in six of seven studies. Studies involving cohorts in the United Kingdom, China and Spain found that dementia was associated with severe but not milder depression.^23^, ^37^ Dose response relationships between depression severity and dementia were found in a cohort study involving 14 countries (*p* for trend <0.001),^38^ and a Canadian cohort study.^39^ In Swedish register data, dementia was associated with depression at all severity levels, although the association only remained significant for longer than 10 years in moderate and severe depression.^24^ In a cohort of men in Western Australia, both severe and mild‐to‐moderate depression were associated with dementia.^35^

In a register‐based study from Taiwan which adjusted for comorbid alcohol and substance‐related disorders, the strongest association between depression and dementia was found in difficult‐to‐treat patients who had failed to respond to at least two antidepressant trials (HR: 5.19, 95% CI: 2.56–10.52), with a less pronounced association in those with no antidepressant prescription (HR: 2.37, 95% CI: 1.87–3.01).^40^ A cohort study in Western Australia found that the association between depression and dementia did not vary by anti‐depressant medication use,^35^ whereas a study conducted in Spain found that untreated depression, but not depression treated with anti‐depressants, was associated with increased risk of AD, although associations attenuated in both groups after adjustment for confounders.^37^ Associations between depression and dementia remained present in studies which conducted competing risks regression to account for differing patterns of mortality between groups.^35^, ^37^, ^38^

### 3.4 Anxiety

We identified seven longitudinal studies conducted in Europe examining associations between anxiety and subsequent dementia (Table 1). In six prospective cohort studies, anxiety was assessed via questionnaires or self‐report measures, while a Finnish register‐based study used ICD‐8 diagnoses.^33^ Sample sizes ranged from 1082^41^ to 55,896,^33^ and study quality was good (*n* = 5) or poor (*n* = 2). Mean follow‐up time was between 4.4^42^ to 19.4 years,^33^ and four of seven studies had follow‐up periods longer than 10 years.^7^, ^33^, ^41^, ^43^

Findings were mixed, with four studies finding an association between anxiety and dementia. Associations were observed in a Swedish cohort study with a mean time of 14.7 years to dementia (HR: 1.48, 95% CI: 1.01–2.18),^41^ and in the Caerphilly Prospective Study, with a mean follow‐up of 17.3 years (OR: 2.89, 95% CI: 1.27–6.54),^43^ including after adjusting for depression. Petkus et al. found that anxiety, but not depression, showed an independent association with dementia.^41^ An association was found between anxiety and dementia in the Three‐City Study in France (HR: 1.26, 95% CI: 1.01–1.57), although this attenuated to the null after adjusting for depression symptoms (HR: 1.04, 95% CI: 0.81–1.32).^44^ In a prospective cohort study in Spain which incorporated mortality as a competing risk and adjusted for depression, clinically‐significant anxiety (SHR: 3.9, 95% CI: 1.59–9.6), but not subthreshold anxiety (SHR: 1.19, 95% CI: 0.75–1.88), was associated with AD over 4.5 years ^42^

Conversely, a prospective cohort study in the Netherlands with 18 years of follow‐up,^7^ and a Finnish register‐based study with up to 33 years of follow‐up, found no association between anxiety and dementia regardless of the interval between diagnoses.^33^ A Spanish cohort study with 4.5 years of follow‐up found an association between anxiety and VaD among men approaching significance (SHR: 2.61, 95% CI: 0.88–7.74), but not among women (SHR: 0.7, 95% CI: 0.25–1.99) using Fine and Grey competing risks regression and after adjustment for depression.^45^ We identified five prospective cohort studies, conducted in the Netherlands, Wales, France and Sweden, providing comparable estimates which could be pooled in a meta‐analysis. Sample sizes ranged from 1,082^41^ to 55,896^33^ and follow‐up periods were between 10^44^ and 33 years.^33^ All studies were assessed as good quality. We found a pooled RR of 1.18 (95% CI: 0.96–1.45; *I* ^2^ = 52.2%) for the association between anxiety and subsequent dementia (Figure 2).

### 3.5 PTSD

We identified three population‐based cohort studies examining associations between PTSD and all‐cause dementia in the Unites States, Denmark and Taiwan (Table 1). ICD‐9/10 diagnoses of PTSD and dementia were obtained from medical records. Sample sizes ranged from 8750^46^ to 499,844,^47^ and total follow up periods were 11,^46^ 13^47^ and 17 years,^48^ respectively. Although it was not possible to conduct quantitative synthesis due to an insufficient number of citations, all identified studies observed associations between PTSD and dementia (HR: 1.70 [95% CI: 1.45–2.00], HR: 2 [95% CI:1.3–3.2],^48^ HR: 4.37 [95% CI: 2.53–7.55]).^46^

In routine data from California, the association was found in both men (HR: 1.96, 95% CI: 1.51–2.55) and women (HR: 1.59, 95% CI: 1.3–1.95), with a more pronounced association among those with comorbid depression.^47^ In register data from Taiwan, associations were found in women (HR: 4.8, 95% CI: 2.52–9.12) and men (HR: 3.33, 95% CI: 1.22–9.06).^46^ A dose‐response relationship was found between dementia and PTSD severity, indexed by frequency of visits to psychiatric clinics per year for PTSD (<5 visits HR: 2.81, 95% CI: 1.5–5.25; >10 visits HR: 18.13, 95% CI: 9.13–36.00).^46^ Associations remained present after adjusting for depression, and two studies found that the relationship between PTSD and dementia was stronger among those with comorbid depression.^46^, ^47^

### 3.6 Bipolar disorder

We identified five studies examining associations between bipolar disorder and dementia (Table 1). Two studies ascertained diagnoses from the Western Australian Data Linkage System,^49^, ^50^ and three used register data from Taiwan.^51^, ^52^, ^53^ Sample sizes ranged from 4582^51^ to 102,675,^52^ and follow‐up periods were between 9^53^ and 17.6 years.^49^ All studies were assessed as good quality. Given several studies used the same datasets, it was not possible to pool estimates. Nonetheless, there was consistent evidence of associations between bipolar disorder and dementia. In register data from Taiwan, a strong association was found between bipolar disorder and dementia in men (OR: 4.01, 95% CI: 2.53–6.35) and women (OR: 4.55, 95% CI: 3.07–6.73).^53^ In a cohort in Western Australia, dementia was associated with late‐onset (≥60 years HR: 2.99, 95% CI: 2.17–4.12) and younger‐onset bipolar disorder in men (<60 years HR: 2.31, 95% CI: 1.77–3.01),^49^ and with recent (<5 years HR: 3.23, 95% CI: 2.03–5.14) and long‐standing bipolar disorder (≥15 years HR: 3.09, 95% CI: 2.16–4.43).^50^ Compared to those with bipolar disorder with no psychiatric admissions, the rate of dementia increased by 2.4‐fold in those with 1‐2 psychiatric admissions per year (95% CI: 1.85–3.11) versus 5.72‐fold for those with >2 admissions (95% CI: 4.8–6.81),^52^ suggesting that the association may vary by severity. Associations between bipolar disorder and dementia were consistent in studies using Fine and Grey models to examine the competing risk of mortality,^50^, ^52^ and remained present in studies which adjusted for alcohol and substance‐related disorders.^49^, ^50^, ^51^, ^53^

### 3.7 Psychotic disorders

Nine studies conducted in Australia, Asia, Europe and North America reported associations between psychotic disorders and dementia (Table 1). Sample sizes ranged from 12,616^54^ to 8,011,773,^22^ and follow‐up periods were between 4^55^ to 33 years.^33^ Studies were good (*n* = 8) or poor (*n* = 1) quality. ICD‐8‐10 diagnoses were obtained from medical records. Seven studies, conducted in Australia, Israel, Taiwan, Denmark, Sweden, and Finland, provided comparable estimates to pool in a meta‐analysis, demonstrating an association between psychotic disorders and dementia (pooled RR: 2.19, 95% CI: 1.44–3.31; *I* ^2^ = 99%; Figure 2). Two further studies, which could not be pooled in the meta‐analysis due to a lack of comparable data, also found associations between psychotic disorders and dementia. Using routine data from the United States, Stroup et al. found that schizophrenia was associated with incident dementia across a wide range of ages,^22^ and Kessing et al.^56^ found an association between schizophrenia and dementia in Danish register data (SMR: 14.7, 95% CI: 9.1–22.4).

All four of the studies focussed specifically on very late‐onset schizophrenia‐like psychosis (VLOSLP) observed associations with dementia,^20^, ^54^, ^55^, ^57^ with HRs ranging from 2.21 (95% CI: 1.39–3.5)^54^ in Danish register data to 4.22 (95% CI: 4.05–4.41) in Swedish register data.^20^ In a Western Australian cohort, the association between psychotic disorders and dementia was stronger with a shorter duration of psychosis (<5 years HR: 4.25, 95% CI: 2.71–6.67; ≥10 years HR: 2.42, 95% CI: 2.10–2.8).^57^ Similarly, in Swedish register data the association between psychotic disorders and dementia was strongest in the first year following VLOSLP diagnosis and attenuated over time, despite remaining present for up to 20 years of follow‐up.^20^ Patterns of association between psychotic disorders and dementia remained consistent in studies which used competing risks regression to examine whether findings could be explained by differing patterns of mortality between groups.^20^, ^54^, ^55^, ^57^

### 3.8 PAFs

Using pooled RRs obtained in this review and prevalence estimates of psychiatric disorders from the APMS, we estimated PAFs, indicating the proportion of dementia cases theoretically preventable through elimination of psychiatric disorders in the population, assuming associations were causal: depression: 3.1%, anxiety: 1.1%, and psychotic disorders: 0.8% (Table 3).

**TABLE 3:** Population attributable fractions (PAF)

Psychiatric disorder	Prevalence (%)^a^	RR^b^	PAF
Depression	3.3	1.96	3.07
Anxiety	5.9	1.18	1.05
Psychotic disorder	0.7	2.19	0.83

Abbreviations: PAF, population attributable fraction, RR, relative risk;

^a^ Prevalence estimates obtained from the Adult Psychiatric Morbidity Survey.

^b^ RRs obtained from pooled estimates in systematic review.

## 4 DISCUSSION

### 4.1 Summary of findings and comparison with previous literature

We found that depression, PTSD, bipolar disorder and psychotic disorders were associated with increased risk of subsequent all‐cause dementia. Building on previous meta‐analyses,^3^, ^4^ we focused only on longitudinal, population‐based studies and clinically‐significant psychiatric symptoms or diagnoses. Findings were similar in studies which took mortality into account as a competing risk, increasing confidence that the results were not attributable to selective attrition. Associations between depression and dementia were stronger in studies with shorter follow‐up periods, where the interval between diagnoses was shorter, and for severe and late‐onset depression, suggesting that the association may at least partly reflect reverse causation. Few studies examined the role of psychiatric comorbidities, although there was some evidence that comorbid depression was associated with increased risk of dementia in PTSD.^46^, ^47^

Importantly, most identified studies focused on depression, whereas the evidence‐base was considerably weaker for other psychiatric disorders, limiting the possibility for quantitative synthesis. Nonetheless, we found that psychotic disorders were associated with increased risk of dementia, with narrative evidence of a stronger association when the interval between diagnoses was shorter. In contrast, we did not observe an overall association between anxiety and dementia. This corresponds with a previous meta‐analysis which did not find a clear association in analyses including only adjusted estimates (pooled RR: 1.68, 95% CI: 0.94–3.02).^4^ However, the review included studies with unrepresentative samples and participants with MCI at baseline, and all except one study had follow‐up periods shorter than 3.8 years. Our finding of an association between PTSD and dementia corresponds with results from a previous review (HR: 1.61, 95% CI: 1.43–1.81),^8^ while another review did not find evidence of a significant pooled association (OR: 2.55, 95% CI: 0.43–15.12).^9^ In contrast, our review only included studies focused on the general population, rather than veteran or clinical samples.

### 4.2 Strengths and limitations

Strengths include pre‐registration, carefully defined eligibility criteria to reduce selection bias, and a comprehensive search strategy. In contrast to previous reviews, we focused on longitudinal, population‐based epidemiological studies, and clinically significant psychiatric symptoms or diagnoses. We also note several limitations. Our strict eligibility criteria resulted in relatively few citations, limiting our ability to conduct quantitative syntheses and subgroup analyses. In addition, our focus on clinical psychiatric diagnoses may have favoured retrospective studies of routine data and resulted in exclusion of prospective cohort studies. In addition, retrospective studies tend to have larger sample sizes and therefore often receive larger weights in meta‐analyses. This may have influenced results in that retrospective studies tend to have longer follow‐up periods and may be more prone to biases such as exposure and outcome misclassification and residual confounding. Nonetheless, subgroup analyses for depression did not indicate significant differences in the association between depression and dementia for prospective versus retrospective studies, although it was not possible to examine these differences for other psychiatric diagnoses due to the small number of identified studies. Given high levels of heterogeneity between estimates, pooled estimates should be interpreted cautiously and alongside narrative synthesis. Heterogeneity was lower for AD and VaD outcomes, indicating more consistent effect sizes compared with the all‐cause dementia outcome, where higher heterogeneity could partly reflect differing proportions of AD and VaD across different samples. Limiting our search to English language papers could have led to exclusion of relevant papers in other languages. Only half of included studies had follow‐up periods longer than 10 years, although our pre‐planned subgroup analyses allowed us to consider the effect of this limitation. Further, most studies did not report mean follow‐up time, or interval between diagnoses, limiting our ability to examine temporal relationships.

### 4.3 Meaning of findings

Associations between psychiatric disorders and dementia are compatible with several explanations. First, psychiatric disorders are associated with poor cardiovascular health^58^, ^59^, ^60^ and negative health behaviours,^61^, ^62^, ^63^ which could, in turn, increase risk for dementia.^64^, ^65^, ^66^ Systemic inflammation and cerebrovascular disorder have been implicated in the pathophysiology of dementia^67^, ^68^ and psychiatric disorders,^69^, ^70^ and has been suggested as a candidate pathway in explaining these relationships.^71^ Associations could be driven by cognitive reserve,^72^ as some individuals with psychiatric disorders could have lower levels of baseline cognitive functioning and may therefore require less neuropathology before meeting clinical thresholds for dementia diagnosis.^73^ Psychiatric disorders are associated with high levels of stress^74^, ^75^ and it is possible that related neurobiological pathways, including hypothalamic‐pituitary‐adrenal axis dysfunction, could increase risk of dementia.^76^, ^77^ This is an important area for future research, given that few studies have directly tested these potential mechanisms.

Conversely, it is possible that, rather than risk factors, psychiatric symptoms could represent early manifestations of AD neuropathology, which begins to accumulate in the brain over several decades before cognitive and functional decline are apparent.^11^ Correspondingly, we found stronger associations between dementia and depression in studies with shorter follow‐up periods, and for late‐onset depression. Another potential explanation is that people with psychiatric disorders have more contact with clinical services,^78^ allowing easier detection of dementia, whereas missed or delayed diagnoses may be more likely in those without psychiatric illness. However, our exclusion of clinical samples reduced the likelihood of this measurement error affecting our results.

Our findings demonstrate that patients with psychiatric disorders are at increased risk for dementia, highlighting the importance of ongoing monitoring for functional and cognitive decline in these groups. Importantly, our results demonstrate a need for further high‐quality longitudinal evidence, particularly regarding anxiety, PTSD and bipolar disorder, where evidence was sparse or mixed. Further, studies examining patterns of association over time are required to establish whether associations are present up to 20–30 years before dementia, where bias due to preclinical neurodegeneration is small, or only emerge close to dementia diagnosis, consistent with reverse causation. Studies should also investigate the effect of treatment of psychiatric disorders on dementia risk, given the potential implications for clinical practice and public health. In addition, further research is needed to examine the role of psychiatric comorbidities, which are pervasive and were found to be associated with increased risk of dementia in PTSD. Establishing whether psychiatric disorders are causal risk factors for dementia would have important implications for dementia prevention and treatment and management of psychiatric disorders in mid‐ and late‐life.

## CONFLICT OF INTEREST

None.

## Supporting information

Supplementary Material

## Data Availability

The data that support the findings of this study are available from the corresponding author upon reasonable request.
